# Candidate Proficiency Test Chemicals to Address Industrial Chemical Applicability Domains for *in vitro* Human Cytochrome P450 Enzyme Induction

**DOI:** 10.3389/ftox.2022.880818

**Published:** 2022-06-20

**Authors:** Miriam Naomi Jacobs, Barbara Kubickova, Eugene Boshoff

**Affiliations:** Centre for Radiation, Chemical and Environmental Hazards (CRCE), Department of Toxicology, Public Health England (PHE), Harwell Science and Innovation Campus, Chilton, United Kingdom

**Keywords:** CYP, P450, validation, test guideline, HepaRG, metabolism

## Abstract

Cytochrome P450 (CYP) enzymes play a key role in the metabolism of both xenobiotics and endogenous chemicals, and the activity of some CYP isoforms are susceptible to induction and/or inhibition by certain chemicals. As CYP induction/inhibition can bring about significant alterations in the level of *in vivo* exposure to CYP substrates and metabolites, CYP induction/inhibition data is needed for regulatory chemical toxicity hazard assessment. On the basis of available human *in vivo* pharmaceutical data, a draft Organisation for Economic Co-operation and Development Test Guideline (TG) for an *in vitro* CYP HepaRG test method that is capable of detecting the induction of four human CYPs (CYP1A1/1A2, 2B6, and 3A4), has been developed and validated for a set of pharmaceutical proficiency chemicals. However to support TG adoption, further validation data was requested to demonstrate the ability of the test method to also accurately detect CYP induction mediated by industrial and pesticidal chemicals, together with an indication on regulatory uses of the test method. As part of “GOLIATH”, a European Union Horizon-2020 funded research project on metabolic disrupting chemical testing approaches, work is underway to generate supplemental validated data for an additional set of chemicals with sufficient diversity to allow for the approval of the guideline. Here we report on the process of proficiency chemical selection based on a targeted literature review, the selection criteria and considerations required for acceptance of proficiency chemical selection for OECD TG development (i.e. structural diversity, range of activity, relevant chemical sectors, global restrictions etc). The following 13 proposed proficiency chemicals were reviewed and selected as a suitable set for use in the additional validation experiments: tebuconazole, benfuracarb, atrazine, cypermethrin, chlorpyrifos, perfluorooctanoic acid, bisphenol A, N,N-diethyl-m-toluamide, benzo-[a]-pyrene, fludioxonil, malathion, triclosan, and caffeine. Illustrations of applications of the test method in relation to endocrine disruption and non-genotoxic carcinogenicity are provided.

## Introduction

The application of *in vitro* test method tools for chemical hazard assessment in relation to human health protection is often limited due to insufficient understanding of chemical metabolism, bioactivation or deactivation, and bioavailability.

The liver is a main site of Phase I and Phase II metabolism of endogenous and exogenous substances including nutrients, drugs and chemicals, but many other tissues (such as but not limited to the gut, kidney, placenta) in the body do also have metabolism competency. Phase I metabolism encompasses the biochemical reactions that introduce reactive and polar groups into xenobiotic compounds by oxidation, reduction, or hydrolytic reactions. The major Phase I modifications are catalysed by a large family of Cytochrome P450 (CYP) enzymes (Lewis 2002), and in the first steps, CYPs may transform a xenobiotic into a harmless metabolite (detoxification) that can be easily eliminated via Phase II, or, vice versa, a non-toxic parent chemical may be transformed (metabolically bioactivated) into a toxic metabolite.

CYP induction, that is transcriptional activation/upregulation in CYP gene expression and protein levels, is first triggered by the binding of a chemical to specific nuclear receptors, these constitute the molecular initiation event (MIE). The Aryl hydrocarbon Receptor (AhR), is primarily responsible for the CYP1A and 1B family, the Pregnane X Receptor (PXR) for the CYP3A family and the Constitutive Androstane Receptor (CAR) for the CYP 2B family (Waxman 1999), and the Peroxisome Proliferator Activated receptors (PPARs) for the CYP4A family. These receptors also play major roles in regulating many physiological functions, including hormone and lipid regulation (Kliewer, Lehmann et al., 1999; Jacobs and Lewis 2002).

The CYP1A, 1B, 3A and 2E subfamilies are responsible for the bioactivation of the majority of xenobiotics. For example, with repeated exposure, many chemical carcinogens are bioactivated by CYP1A, indeed such chemicals selectively induce this family, thus exacerbating their carcinogenicity (Ioannides and Lewis 2004; Rendic and Guengerich 2021). Pharmaceuticals, nutrients and some industrial chemicals are mostly activated by CYP3A4, and dietary nutrients/contaminants by CYP1A2, 2E1, and 3A4 (Jacobs and Lewis 2002; Jacobs, Nolan et al., 2005; Hakkola, Hukkanen et al., 2020; Rendic and Guengerich 2021 and references therein). Endogenous substances that are usually components of physiological processes, are mainly activated by CYP1A, 1B1, and 3A enzymes (Rendic and Guengerich 2021). The latter recent review analysing the catalytic activity of CYP families in relation to catalytic bioactivation showed predominant participation of CYP3A4, 1A2, and 1A1, followed by CYP2E1 and 1B1. CYPs 2C9, 2D6, 2A6, 2C19, and 2B6 also having significant participation.

Phase II metabolism often involves the further conjugation of the metabolite with polar molecules, such as sulphate, amino acids, glutathione or glucuronic acid, facilitated by various transferases, generating metabolites that are more soluble to facilitate elimination.

Phase I CYP enzyme induction therefore plays a pivotal initial role in the metabolism of both xenobiotics and endogenous chemicals and constitutes a sensitive biomarker for metabolic competence of *in vitro* test systems. Chemically mediated induction and/or inhibition of CYPs can lead to marked changes in CYP substrate and metabolite concentrations, and *in vitro* CYP induction and inhibition data are currently commonly used to predict potential CYP mediated clinical drug interactions for pharmaceuticals (EMA 2012; US FDA, 2020) and are compiled in pharmaceutical CYP induction/inhibition databases (e.g. SIMCYP (Marsousi, Desmeules et al., 2018)).

Thus CYP induction and inhibition data are also needed for a wide number of human health endpoints both for pharmaceutical therapeutic and chemical hazard assessment purposes, for instance ranging from hormone and fatty acid metabolism (Kliewer, Lehmann et al., 1999), hepatotoxicity, and steatosis (Massart, Begriche et al., 2022) to, inflammatory responses (Rubin, Janefeldt et al., 2015), and non-genotoxic carcinogenicity (Ioannides and Lewis 2004; Jacobs, Colacci et al., 2016).

Furthermore, the greater inclusion of CYP induction/inhibition data into chemical hazard assessment will facilitate the shift from regulatory reliance on animal *in vivo* testing to New Approach Methodologies (NAMs) that refer to and include a battery of relevant *in vitro* and *in silico* tools. While the incorporation of metabolic capacity into *in vitro* genotoxicity testing has been routinely conducted for several decades, in contrast, although discussed a lot, it has not progressed very quickly for other human health endpoints. Illustrations of how the tests could be combined within the OECD conceptual frameworks and Integrated Approaches to Testing and Assessment (IATA) have been developed (Jacobs, Laws et al., 2013; Jacobs, Colacci et al., 2020).

Whilst many chemicals are metabolized by CYPs, enzyme induction data are the focus of the HepaRG CYP enzyme induction test method (Bernasconi, Pelkonen et al., 2019). The need for human CYP induction/inhibition test data to improve the predictive accuracy of *in vitro* test methods and *in silico* tools for chemical toxicological hazard assessment, was established by the OECD Test Guideline Programme over 14 years ago, particularly in relation to endocrine disruption (Jacobs, Janssens et al., 2008; OECD 2008). OECD member countries recommended that the human CYP enzyme induction test method was the optimum metabolism test method to take forward for OECD Test Guideline (TG) purposes, and on this basis, validation activities were later initiated and completed for a test method for an *in vitro* human hepatocyte cell line CYP enzyme induction assay. The assay is capable of detecting an induction of the enzymatic activity of four CYP isoforms (CYP1A1/1A2, 2B6, and 3A4) (Bernasconi, Pelkonen et al., 2019), and was developed and validated using pharmaceutical chemicals. These CYP enzymes are commonly involved in metabolizing drugs and environmental toxicants (Esteves, Rueff et al., 2021), and in producing pharmacokinetic (PK) interactions of medicines (EMA 2012; Ooka, Lynch et al., 2020; US FDA, 2020).

The test method utilizes the metabolic capacity of differentiated cryopreserved immortalized human HepaRG cells coupled with analytical liquid chromatography and mass spectrometry (LC-MS) to quantify the induction of the CYP enzymes based on model substrate conversion (Bernasconi, Pelkonen et al., 2019). To improve human relevance, the study was designed on the basis of human CYP induction evidence, rather than the more plentiful *in vivo* rodent data. There was sufficient relevant human CYP induction data to allow for an assessment of the human translation potential of the test method, available only for pharmaceuticals (JRC TSAR, 2009). At that time there were no human *in vivo* data available for other regulatory sector classes (not least because there are ethical issues with respect to pesticide and contaminant testing in controlled human studies). In 2019, a draft TG was submitted for review to the OECD that was based upon the successfully performed and peer reviewed validation data generated using pharmaceutical chemicals. Review feedback was received from the OECD Working Group of National Coordinators of the TG Programme (WNT) that an essential requirement for the approval of the draft TG would be the provision of supporting validation data generated with additional proficiency chemicals representative of chemicals used in other relevant sectors, including industrial chemicals and pesticides. This was because some members of the WNT did not consider that the chemical applicability domain of pharmaceuticals tested in the original validation, gave sufficient coverage of the industrial chemical applicability domain that this test method is intended to be applied to. Due to the lack of primary human data for non-pharmaceuticals, the WNT accepted a compromise proposal on how to utilise the wider chemical metabolism data in the scientific literature, using metabolism data generated from relevant human cell lines, for example. This data was utilised to generate a chemical selection list, to supplement the reference/proficiency chemical list in the original HepaRG CYP enzyme induction test method validation, and the review and selection process is described in this paper. This list of suitable proficiency chemicals is the basis for augmenting the chemical applicability domain of the test method. This additional validation data is being generated on the HepaRG CYP enzyme induction test method, within “GOLIATH”, a European Union Horizon-2020 funded research project on metabolic disrupting chemical testing approaches.

Here we provide details on the selected chemicals, the method and supporting data used for selecting the chemicals, and an overview of intended regulatory applications of the test method.

## Methodology

### Criteria Used for the Identification of an Initial Selection Pool of Candidate Validation Chemicals

The chemical and structural diversity of the proficiency chemicals used for (pre)validation needs to address both the chemical applicability domain of the chemical Universe for which the test method is intended to predict endpoint-specific toxicity, but also be structurally relevant for the biological role of the endpoint. In addition, in many cases, where known, natural and endogenous ligands should be included in the chemical selection (Waxman 1999; Jacobs and Lewis 2002) as these are the ligands that the anthropogenic chemicals of concern are mimicking.

A targeted reiterative but not systematic literature search was carried out to identify an initial selection pool of candidate proficiency chemicals for which human CYP modulation data was available for CYP1A1/1A2, CYP2B6, and CYP3A4, and that belonged to OECD TG Programme relevant chemical classes (including industrial chemicals, pesticides, and food additives). Data sources from both human cell line *in vitro* and human *in vivo* studies relating to specified CYP induction and directly related receptors were sourced and critically evaluated, but due to the general scarcity of *in vivo* human studies carried out with non-pharmaceutical chemicals, only human *in vitro* data was available for most chemicals. Support that this would be an acceptable approach to take was first established within the OECD WNT, given the scarcity of human *in vivo* data. In some cases, it was appropriate to use rodent data for weight of evidence support.

On this regulatory acceptable basis, between 2017 and 2021, Scopus and pubmed search engines were queried with respect to human relevant data, cytochrome P450, and chemicals, including pesticides/bacteriocides and excluding pharmaceuticals as these are already addressed in the draft TG. The results were filtered and prioritised for human relevant data including human relevant cell lines, e.g. HepaRG, HepG2, and the relevant CYPs in the draft validated TG, together with relevant references within the papers. These were critically reviewed and double checked by the authors and then external regulatory experts for the OECD TG Programme (WNT).

In the validated test method, overall CYP enzymatic activity is quantified in an *in vitro* human hepatocyte cell line (cryopreserved HPR116 differentiated HepaRG cells) before and after pretreatment with test chemicals, by measuring the rate of metabolic conversion of substrates that are selective for CYP1A1/1A2 (phenacetin to acetaminophen), CYP2B6 (bupropion to hydroxybupropion), and CYP3A4 (midazolam to 1′-hydroxymidazolam) using a LC-MS analytical technique (Bernasconi, Pelkonen et al., 2019). In such cell systems, the overall effect on enzyme activity is dependent on the extent to which a chemical up or downregulates not only CYP mRNA/protein levels, but also functional enzymatic activity. Data evaluated in the literature search therefore included *in vitro* mRNA/protein quantity and enzyme activity data generated using human cells, as well as enzyme activity data generated in human liver microsomes (HLMs) and recombinant enzyme preparations (REPS). CYP1A1/1A2 gene expression is well understood and extensively documented to be induced by activation of the AhR (e.g. Bock 2014; Vogel, Van Winkle et al., 2020), and activation of CAR and PXR has been shown to induce CYP2B6 and CYP3A4 (Tolson and Wang 2010; Wang, Ong et al., 2012). Data showing activation of these receptors was taken to indicate likely mRNA/protein upregulation of the respective CYPs. Preliminary assessment as to whether a candidate chemical is likely to induce, inhibit, or have no effect on a specific CYP in the test method was made on the basis of enzyme activity data from human cell systems with innate CYP expression, when this data was available. Where this data was not available, estimations were made based on available mRNA/protein quantity data and enzyme activity data from non-cell preparations, when possible. In the latter case, estimations were considered to be of lower reliability as compared to cases where cell-based enzymatic activity data was available, and insufficient data were considered to be available to estimate the effect of a chemical in the test method if overall the available good quality data was considered to be contradictory. In the design of validation experiments intended for applications beyond classification and prioritisation purposes, it is good practice to include proficiency chemicals that are expected to produce a potency range from negative to low, moderate, and strong effects, and to generate concentration-response information, as this is of greater utility for IATA approaches (Jacobs, Ezendam et al., 2022). Thus, for chemicals for which cell-based enzymatic activity data showed them to be an inducer, the magnitude of any observed CYP enzyme induction was categorized as low (≤3 fold), moderate (>3 to 4.5 fold), or strong (>4.5 fold), and this information was used in the chemical selection considerations. When cell-based enzymatic data was absent (not tested), the magnitude of an expected effect was categorized as uncertain.

As the conversion of phenacetin to acetaminophen is catalyzed by both CYP1A1 and 1A2 (Kcat = 0.84 and 2.2 min^−1^, respectively) (Huang, Deshmukh et al., 2012), production of acetaminophen is used as an overall marker of both CYP1A1 and 1A2 activity in the CYP enzyme induction assay; and CYP1A1/1A2 activity predictions were thus made based on the cumulative available data for both CYP1A1 and 1A2. Formation of 1′-hydroxymidazolam is widely used as a selective marker of CYP3A4 activity. The latter reaction is catalyzed to a significant extent by both CYP3A4 and 3A5 (Vmax = 35 and 72 nmol/min/nmol CYP, and Km = 5 and 14 μM, respectively) (Williams, Ring et al., 2002), but the HepaRG cell line contains two CYP3A5*3 alleles, which are known to be null due to expressed RNA instability (Jackson, Li et al., 2016). It is expected that CYP3A4/5 activity in HepaRG cells is predominantly attributable to CYP3A4 activity. Hydroxybupropion formation, on the other hand, has been shown to be catalyzed nearly exclusively by CYP2B6 (Faucette, Hawke et al., 2000; Hesse, Venkatakrishnan et al., 2000), with other CYP isoforms (including CYP2C19 and 3A4) being involved to only a negligible extent (Faucette, Hawke et al., 2001; Sager, Price et al., 2016).

### International Regulatory Mutual Acceptance of Data Considerations

As it is anticipated that on becoming an OECD TG, the test method will be widely utilised internationally, and will fall under the Mutual Acceptance of Data agreement for the OECD member country regulatory jurisdictions, it is important that proficiency chemicals should be associated with the minimum possible transport, supply and usage restrictions, and accommodate national and international limitations on use. Chemicals that are excessively expensive to procure or that have restricted availability in OECD member countries were therefore avoided when possible. Examples of chemicals with restricted availabilities include all chemicals listed under the Stockholm Convention on Persistent Organic Pollutants (POPs) (http://chm.pops.int/TheConvention/ThePOPs/TheNewPOPs/tabid/2511/Default.aspx, accessed 10 December 2021), and certain classes of controlled substances, such as drugs with abuse potential for example anabolic steroids and cannabinols. Chemical mixtures containing undefined or variable chemical constituents, including (stereo)isomers and racemic mixtures were also avoided when possible, due to the potential for between batch variability. Data and data sources that were considered to be of inadequate quality, due to lack of information regarding the successful establishment of the assay(s), or poor reproducibility (Taxvig 2020; Franzosa, Bonzo et al., 2021), were not utilised.

### Selection of the Validation Chemical Set From the Candidate Selection Pool

From the candidate pool of chemicals, a proposed set of proficiency validation chemicals was selected to enable adequate coverage of structural diversity but also a representative selection of chemicals from relevant sectors (including industrial chemicals, pesticides, and food additives) that was practically possible, on the basis of publicly available scientific literature. Importantly, to be able to fully evaluate the functioning and reliability of the test method, the proposed proficiency chemicals were also chosen to try to ensure the inclusion of a sufficient number of negative chemicals (a minimum of 25% of total tested), with the range of positive chemicals that would adequately probe the ability of the test method to detect individual induction of each of the four measured CYPs (CYP1A1/1A2, CYP2B6, and CYP3A4).

## Results and Discussion

### Proposed Proficiency Chemical Set to Use in Further Validation Experiments

Using a targeted literature search and the selection criteria detailed in the methodology section, an initial pool of potential proficiency candidates consisting of a total of 23 chemicals were identified, and a tabular listing of these chemicals which includes summaries of available CYP activity data and chemical structure images are provided in Table 1, Parts A and B. From these candidates, on the basis of the review exercise, the following 13 chemicals were selected to augment the current proficiency chemical list, consisting of pharmaceuticals and to be proposed as additional proficiency chemicals for further validation experiments: tebuconazole, benfuracarb, atrazine, cypermethrin, chlorpyrifos, perfluorooctanoic acid (PFOA), bisphenol A (BPA), N,N-diethyl-m-toluamide (DEET), benzo-[a]-pyrene (B [a]P), fludioxonil, malathion, triclosan and caffeine (See Table 1 Part A). These 13 chemicals have a diverse range of structures and molecular weights and include representative examples of industrial chemicals, pesticides, and food and cosmetics additives. Excepting caffeine, all the chemicals display a degree of lipophilicity (experimental log P ranging from 1.97 to 7.75). Any potential solubility issues will be evaluated and addressed by solubility and cytotoxicity assessment that are part of the planned validation augmentation study design. These data will be reported following completion of the planned experiments.

**TABLE 1:** Part A = Proposed Set of Additional Industrial, Pesticidal, and Food Additive Proficiency Chemicals to Use in Further CYP Induction Validation Experiments. Part B = Initial Candidate Selection Pool Chemicals that were Evaluated but Not Selected. The magnitude of any observed CYP enzyme induction was categorized as low (≤3 fold), moderate (>3 to 4.5 fold), and or strong (>4.5 fold), when data from cell-based enzymatic activity assays was available. When cell-based enzymatic data was absent (not tested), the magnitude of an expected effect was categorized as uncertain. Data on induction of mRNA or protein was not used to estimate magnitudes of effect.

Chemical name CAS number structure Ause molecular weight (Dalton)^a^Predicted LogP^a^	Expected overall net effect on human CYP enzymatic activity in vitro in HepaRG cells: No effect/Inducer/Inhibitor. For Induction Magnitude = + Weak, ++ Moderate, +++ Strong, ∼ uncertain. Na = No or insufficient data			Supporting evidence from literature publications
	CYP1A1/1A2	CYP2B6	CYP3A4	
Part A. Proposed set of additional proficiency chemicals				
**Tebuconazole**;107534-96-3 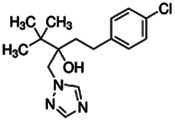 ; Triazole fungicide used on plants; 307.818;3.58	Inducer (+)	Inducer (+)	Inducer (+)	**CYP1A1:** In HepaRG cells, 80 µM increased mRNA ≤4 (log_2_)x [ = ≤16.0x] Abass and Pelkonen (2013), Lasch, Marx-Stoelting et al. (2021), and protein 3.25 (log_2_)x [ = 9.5x] Schmidt, Lichtenstein et al. (2021). In Caco-2 cells, 8.1 µM weakly increased enzyme [ethoxyresorufin-O-deethylase (EROD)] activity (∼7 pmol/min/mg protein vs 0 for the control) Sergent, Dupont et al. (2009). **CYP1A2**: In HepaRG cells, 1.25-40 µM increased mRNA ≤30x and enzyme activity ≤2.5x [ = ≤6.7x] Knebel, Heise et al. (2019), Lasch, Marx-Stoelting et al. (2021), and 80 µM increased protein ≤2.75 (log_2_)x [ = 6.8x] Braeuning, Mentz et al. (2020), Schmidt, Lichtenstein et al. (2021).**CYP2B6:** In HepaRG cells, 5–40 µM increased mRNA and enzyme activity ≤2.5 × Knebel, Heise et al. (2019), Knebel, Neeb et al. (2018), Lasch, Marx-Stoelting et al. (2021), and 80 µM increased protein 1.72 (log_2_) [ = 3.3x] Schmidt, Lichtenstein et al. (2021).**CYP3A4:** In HepaRG cells, 1.25-40 µM increased mRNA ≤6x, and enzyme activity ≤2.5 × Knebel, Heise et al. (2019), Knebel, Neeb et al. (2018), Lasch, Marx-Stoelting et al. (2021); and 80 µM increased protein ≤0.64 (log_2_)x [ = ≤1.6x] Braeuning, Mentz et al. (2020), Schmidt, Lichtenstein et al. (2021). In Caco-2 cells, 8.1 µM increased enzyme activity ∼40% Sergent, Dupont et al. (2009).**AhR, CAR, PXR:** Activated AhR Knebel, Heise et al. (2019) and PXR Knebel, Neeb et al. (2018), and inhibited CAR Knebel, Neeb et al. (2018).**Human Exposure Data:** Urine metabolite (hydroxy-tebuconazole) PK data from 6 volunteers after a single oral (1.5 mg) or dermal (2.5 mg) dose Oerlemans, Verscheijden et al. (2019), and urine PK data from 7 agricultural workers after occupational exposure Fustinoni, Mercadante et al. (2014).
**Benfuracarb;** 82560-54-1 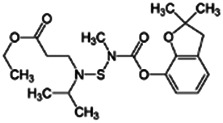 ; Carbamate insecticide; 410.528; 4.54	Inducer (+)	Inducer (+)	Inducer (+)	**CYP1A2:** In HepaRG cells, 10/50 µM increased mRNA and enzyme activity ≤2 × Abass, Lämsä et al. (2012).**CYP2B6:** In HepaRG cells, 10/50 µM increased mRNA ≤5x and enzyme activity ≤3 × Abass, Lämsä et al. (2012).**CYP3A4:** In HepaRG cells, 10/50 µM increased mRNA ≤9x and enzyme activity ≤2.5 × Abass, Lämsä et al. (2012). In human liver microsomes (HLM), inhibited formation of 1-OH-midazolam and SO_2_-omeprazole with IC_50_s of 14.8 and 24.2 µM, respectively Abass, Reponen et al. (2014). **CAR, PXR:** Activated CAR but not PXR Abass, Lämsä et al. (2012).
**Atrazine**; 1912-24-9; 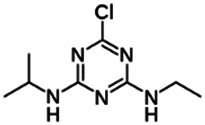 ; Triazine herbicide; 215.683; 2.63	Inducer (+)	Inducer (++)	Inducer (+)	**CYP1A2:** In HepaRG cells, 10/50 µM increased mRNA ≤5x and enzyme activity ≤3 × Abass, Lämsä et al. (2012). In HLM, 1–100 µM did not alter enzyme activity Abass, Lämsä et al. (2012).
				**CYP2B6:** In HepaRG cells, 10/50 µM increased mRNA ≤12x, and enzyme activity ≤4 × Abass, Lämsä et al. (2012). In HLM, weakly inhibited enzyme activity (IC_50_ = 107 µM)^**2**^.
				**CYP3A4:** In HepaRG cells, 10/50 µM increased mRNA ≤10x, and enzyme activity ≤2.5 × Abass, Lämsä et al. (2012). In HLM, inhibited formation of midazolam (IC_50_ = 2.8 µM) but not SO_2_-omeprazole (IC_50_ = 618 µM) Abass and Pelkonen (2013).
				**CAR, PXR:** Activated PXR but not CAR Abass, Lämsä et al. (2012).
**Cypermethrin**; 52315-07-8; 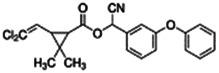 ; Pyrethroid insecticide; 416.297; 6.27	No Effect	Inducer (++)	Inducer (++)	**CYP1A1:** In HepG2 and HaCat cells, 100 µM had no effect on enzyme (EROD) activity Delescluse, Ledirac et al. (1998). In HLM, did not alter CYP1A1/1A2 enzyme activity Abass, Turpeinen et al. (2009).
				**CYP1A2:** In HepaRG cells, 10/50 µM increased mRNA ≤3x, but had no effect on enzyme activity Abass, Lämsä et al. (2012). In HLM, 1–100 µM did not alter enzyme activity Abass and Pelkonen (2013).
				**CYP2B6:** In HepaRG cells, 10/50 µM increased mRNA ≤7x and enzyme activity ≤3.5 × Abass, Lämsä et al. (2012). In primary human hepatocytes (PHH), 10 µM increased protein ∼2 × Lemaire, de Sousa et al. (2004). In HLM, 1–100 µM did not alter enzyme activity Abass and Pelkonen (2013).
				**CYP3A4:** In HepaRG cells, 10/50 µM increased mRNA ≤35x and enzyme activity ≤3.5 × Abass, Lämsä et al. (2012). In PHH, 10 µM increased protein ∼2 × Lemaire, de Sousa et al. (2004). In HLM, weakly inhibited formation of 1-OH-midazolam (IC_50_ = 70 µM) and SO_2_-omeprazole (IC_50_ = 249 µM) Abass and Pelkonen (2013).
				**CAR, PXR:** Activated CAR and PXR Abass, Lämsä et al. (2012), Abass and Pelkonen (2013).
**Chlorpyrifos**; 2921-88-2; 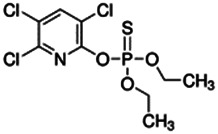 ; Acetylcholinesterase (AChE) Inhibitor Organophosphate pesticide; 350; 4.77	Inducer (+)	Inhibitor	Inducer (+++)	**CYP1A1:** In PHH, 100 µM increased mRNA ≤25x; and 1/10 µM but not 50/100 µM increased enzyme activity ≤2 × Das et al. (2008a).
				**CYP1A2:** In PHH, 100 µM increased mRNA ≤8 × Das et al. (2008a). In HepaRG cells, 10/50 µM increased mRNA ≤5x and enzyme activity ≤3 × Abass, Lämsä et al. (2012). In REPS, inhibited enzyme activity with an IC_50_ of 2.9 µM Abass and Pelkonen (2013).
				**CYP2B6:** In PHH, 100 µM had no effect on mRNA Das et al. (2008a), and 10 µM increased protein ∼2 × Lemaire, de Sousa et al. (2004). In HepaRG cells, 10/50 µM increased mRNA ≤4x, but reduced enzyme activity ∼10 × Abass, Lämsä et al. (2012). In REPS, inhibited enzyme activity with an IC_50_ of 2.5 µM in one study Abass and Pelkonen (2013), and with a Ki of 0.47 µM in another D'Agostino, Zhang et al. (2015).
				**CYP3A4:** In PHH, 100 µM increased mRNA ≤6x, and protein and enzyme activity ∼5 × Das et al. (2008a), and 10 µM increased protein ∼2.5 × Lemaire, de Sousa et al. (2004). In HepaRG cells, 10/50 µM increased mRNA and enzyme activity ≤8 × Abass, Lämsä et al. (2012). In REPS, inhibited 1-OH-midazolam and SO_2_-omeprazole formation with IC_50_s of 4 and 32.2 µM, respectively Abass and Pelkonen (2013).
				**PXR:** Activated PXR Abass, Lämsä et al. (2012), Lemaire, de Sousa et al. (2004).
				**Other CYP Effects:** Inhibited CYP2A6 Abass and Pelkonen (2013), and weakly increased CYP1B1 mRNA Das et al. (2008a). Interspecies differences between rat and human are indicated for CYP2B6 inhibition D'Agostino, Zhang et al. (2015).
				**Human Exposure Data:** Chlorpyrifos use is being phased-out due to it being associated with developmental neurotoxicity in human epidemiological studies at concentrations below the animal lowest observed adverse effect level (LOAEL) for AChE inhibition EFSA (2019). The metabolites of chlorpyrifos are considered unlikely to cause toxicity
**Perfluorooctanoic Acid (PFOA)**; 3825-26-1; 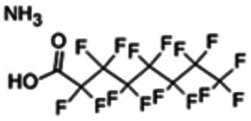 ; Industrial chemical used in non-stick coatings; 414.068; 7.75	Inhibitor	NA	Inhibitor	**CYP1A2:** In HepaRG cells, 1 nM - 1 µM increased mRNA ≥3x at 24 hrs, and at 48 h decreased mRNA ≥5x and enzyme activity ≥1.6 × Franco, Sutherland et al. (2020).
				**CYP2B6:** In HepaRG cells, 30 µM increased mRNA ∼5 fold, whereas 100 µM had no effect on mRNA Abe, Takahashi et al. (2017). In HepG2 cells, 1–100 µM had no effect on mRNA, while 250 µM increased mRNA by 11.2 × Behr et al. (2020b).
				**CYP3A4:** In HepaRG cells, in one publication, 0.1nM-1 µM at 24 h had no effect on mRNA, and at 48 h reduced mRNA ≥10x and enzyme activity ≥5 × Franco, Sutherland et al. (2020); while in another, at 50 and 100 μM, mRNA was increased at 24 and 48 hrs Behr et al. (2020a).
				**CAR, PXR:** Did not activate human PXR or CAR Behr et al. (2020b), Bjork, Butenhoff et al. (2011).
				**Other CYP Effects:** Reduced CYP2C19 mRNA and enzyme activity Franco, Sutherland et al. (2020), and reduced CYP7A1 mRNA and protein Behr et al. (2020a).
				**Additional Info** = PFOA-family compounds with 8–9 carbon backbone have greater activity than PFOA with 7 and 10 carbons. PFOA did not affect cholesterol levels in HepaRG cells but altered bile acid synthesis Behr et al. (2020a), which suggests that PFOA might have cholestatic effects.
				**Human Exposure Data** = Detected at a median concentration of 2.46 ng/ml in human umbilical cords taken from women exposed to air pollutants released by the 2001 September 11 New York World Trade Center attack/collapse Spratlen, Perera et al. (2020).
**Bisphenol A (BPA)**; 80-05-7; 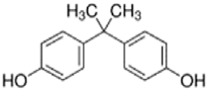 ; Component of certain plastic materials and used as a corrosion inhibitor in metal coatings; 228.286; 3.43	NA	Inhibitor	Inducer (++)	**CYP1A1:** 100 µM increased mRNA in HepG2 cells ∼15x, but had no effect on mRNA in PHH Peyre, Rouimi et al. (2014) **,** Walsky, Astuccio et al. (2006). In recombinant enzyme preparations (REPS), 1 mM inhibited enzyme activity ∼75% Niwa, Tsutsui et al. (2000).
				**CYP1A2:** In PHH, 10/100 µM had no effect on mRNA Vrzal, Zenata et al. (2015). In REPS, 1 mM inhibited enzyme activity ∼60% Niwa, Tsutsui et al. (2000).
				**CYP2B6:** In PHH, 100 µM increased mRNA ≤4 and had no effect on protein Vrzal, Zenata et al. (2015), whereas in HepaRG cells, it reduced mRNA ∼20% Peyre, Rouimi et al. (2014). In REPS, 1 mM inhibited enzyme activity ∼30% Niwa, Tsutsui et al. (2000).
				**CYP3A4:** 100 µM increased mRNA ≤16x in PHH Vrzal, Zenata et al. (2015) and ≤1.3x in HepaRG cells Peyre, Rouimi et al. (2014). In DPX cells, 1–50 µM increased mRNA ≤11x, and increased enzyme activity ≤4x, despite BPA acting as an inhibitor of enzyme activity in HLM (Ki of 57.2 or 43.1 µM) Kuzbari, Peterson et al. (2013). In REPS, 1 mM had no effect on enzyme activity Niwa, Tsutsui et al. (2000).
				**AhR, PXR:** Activated AhR (EC_50_ of 7.9 µM) and PXR (EC_50_s include 6.5 and 11.73 µM) Peyre, Rouimi et al. (2014), Vrzal, Zenata et al. (2015).
				**Human Exposure Data:** Extensive PK data is available from biomonitoring studies and studies in which volunteers were dosed Corrales, Kristofco et al. (2015).
N,N-diethyl-m-toluamide (DEET)**; 134-62-3; 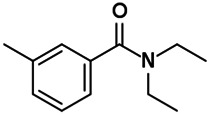 ; Insect repellent; 191.270; 1.96	Inducer (∼)	Inducer (∼)	Inducer (+)	**CYP1A1:** In PHH, 100 µM increased mRNA ≤8 × Das et al. (2008a), Lawrie, Mitchell et al. (2020).
				**CYP1A2:** In PHH, 100 µM increased mRNA ≤4 × Das et al. (2008a) **,** Lawrie, Mitchell et al. (2020). In REPS, 50 µM had no effect on enzyme activity Usmani, Cho et al. (2006).
				**CYP2B6:** In PHH, 100 µM increased mRNA ≤8 × Das et al. (2008a), Lawrie, Mitchell et al. (2020).
				**CYP3A4:** In PHH, 100 µM increased mRNA ≤10x, protein ≤4x, and enzyme activity ∼3 × Das et al. (2008a), Lawrie, Mitchell et al. (2020). In REPS, 50 µM produced a non-significant ∼25% increase in enzyme activity Usmani, Cho et al. (2006).
				**Other CYP Effects:** Induced CYP2A6 Das et al. (2008a).
**Benzo-[a]-pyrene (B**[**a]P**); 50-32-8; 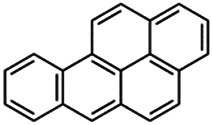 ; Polycyclic aromatic hydrocarbon found in combustion products, for example, in chargrilled food, cigarette smoke, and car exhaust fumes; 252.309; 6.4	Inducer (+++)	NA	NA	**Human Exposure Data:** Human biomonitoring data has shown 2 metabolites of DEET, 3-(diethylcarbamoyl)benzoic acid (DCBA) and 2,3-dihydroxy-4-methoxybenzaldehyde (DHMB), to be present in the urine in a sample of the US general population, with DCBA being detected at geometric mean concentrations ≤4.74 μg/g creatinine ATSDR (2017). Blood and urine PK data is also available for volunteers dermally dosed with [^14^C]-DEET Selim, Hartnagel et al. (1995).
				**CYP1A1:** In HepaRG cells, 5 µM increased mRNA ≤350 × Vlach, Quesnot et al. (2019), and protein 5.1 (log_2_)x [= 34.3x] Schmidt, Lichtenstein et al. (2021), and 10 µM increased mRNA 2.2 (log_2_)x [= 4.6x] Jennen, Magkoufopoulou et al. (2010). In Caco-2 cells, 0.4 µM strongly increased enzyme (EROD) activity (∼40 pmol/min/mg protein vs. 0 for the control) Sergent, Dupont et al. (2009). In REPS, inhibited enzyme activity with an IC_50_ of 0.35 µM Shimada and Guengerich (2006).
				**CYP1A2:** In HepaRG cells, 5 µM increased protein ≤5.3 (log_2_)x [ = ≤39.4x] Schmidt, Lichtenstein et al. (2021). In REPS, inhibited enzyme activity with an IC_50_ of 0.14 µM Shimada and Guengerich (2006).
				**CYP2B6:** In HepaRG cells, 5/10 µM had no effect on mRNA Goedtke, John et al. (2021), Vlach, Quesnot et al. (2019), and increased protein by 1.7 (log_2_)x [= 3.2x] Schmidt, Lichtenstein et al. (2021), and 10 µM decreased mRNA Jennen, Magkoufopoulou et al. (2010).
				**CYP3A4:** In HepaRG cells, 5 µM increased mRNA ∼2.5 × Vlach, Quesnot et al. (2019) and had no effect on protein Schmidt, Lichtenstein et al. (2021). In REPS, inhibited enzyme activity with an IC_50_ of >10 µM Wanchana, Yamashita et al. (2003).
				**AhR, CAR, PXR:** Activated AhR Goedtke, John et al. (2021), weakly activated CAR Goedtke, John et al. (2021), and activated PXR as measured by CYP3A4 promoter activity Luckert, Ehlers et al. (2013).
**Fludioxonil**; 131341-86-1; 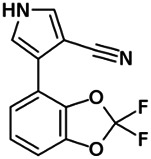 ; Non-systemic phenylpyrrole class fungicide; 248.185; 3.67	Inducer (+++)	Inducer (∼)	Inhibitor	**CYP1A1:** In HepaRG cells, 50 µM increased mRNA strongly and enzyme activity >10 × Lasch, Marx-Stoelting et al. (2021), and 250 µM increased mRNA ≤6x and protein ≤8x (log_2_) [= ≤256.0x] Braeuning, Mentz et al. (2020), Schmidt, Lichtenstein et al. (2021).
				**CYP1A2:** In HepaRG cells, 50 µM increased mRNA ≤250 × Lasch, Marx-Stoelting et al. (2021), and 250 µM increased protein ∼1.8 (log_2_)x [= ∼3.5x] Braeuning, Mentz et al. (2020) **,** Schmidt, Lichtenstein et al. (2021).
				**CYP2B6:** In HepaRG cells, 50 µM increased mRNA ∼4.5 × Lasch, Marx-Stoelting et al. (2021), and 250 µM increased protein ∼1.8 (log_2_)x [= ∼3.5x] Schmidt, Lichtenstein et al. (2021).
				**CYP3A4:** In HepaRG cells, 50 µM had no effect on mRNA Lasch, Marx-Stoelting et al. (2021), and 250 µM decreased protein ∼0.7 (log_2_)x [= ∼1.6x] Braeuning, Mentz et al. (2020), Schmidt, Lichtenstein et al. (2021). In supersomes, 10, 50, and 100 µM inhibited enzyme activity by ∼40%, ∼70%, and ∼75%, respectively Lasch, Marx-Stoelting et al. (2021).
				**AhR, CAR, PXR:** Activated the AhR (EC_50_ = 0.42 µM) and PXR, but not CAR Lasch, Marx-Stoelting et al. (2021), Medjakovic, Zoechling et al. (2014).
**Malathion**; 121-75-5 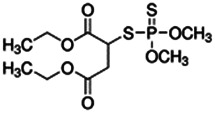 ; Organophosphate insecticide (AChE inhibitor); 330.3582.92	No Effect	No Effect or Inhibitor	NA	**CYP1A2:** In HepaRG cells, 10/50 µM increased mRNA ≤4x, and had no effect on enzyme activity in one publication Abass, Lämsä et al. (2012), while in another, it had no effect on mRNA or enzyme activity Josse, Sharanek et al. (2014). In HLM, inhibited enzyme activity with an IC_50_ of 19 µM Abass and Pelkonen (2013).
				**CYP2B6:** In HepaRG cells, 10–50 µM increased mRNA ≤3x, and decreased enzyme activity ∼95% in one publication Abass, Lämsä et al. (2012) while in another, it had no effect on mRNA or enzyme activity Josse, Sharanek et al. (2014). In HLM, inhibited enzyme activity with an IC_50_ of 69 µM Abass and Pelkonen (2013).
				**CYP3A4:** In HepaRG cells, 10–50 µM increased mRNA ≤4x and enzyme activity ≤2x in one publication Abass, Lämsä et al. (2012), while in another, it had no effect on mRNA or enzyme activity Josse, Sharanek et al. (2014). In HLM, inhibited enzyme activity with an IC_50_ of 57 µM Abass and Pelkonen (2013).
				**CAR, PXR:** Activated CAR but not PXR Abass, Lämsä et al. (2012).
**Triclosan**; 3380-34-5; 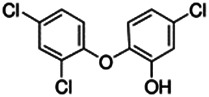 ; Antibacterial and antifungal biocide; 289.542; 5.17	NA	NA	Inducer (∼)	**CYP3A4:** Induced PXR activation in a PXR-CYP3A4 reporter gene assay Jacobs, Nolan et al. (2005). In a human study, everyday exposure to triclosan via toothpaste, which produced 26–296 ng/g plasma concentrations, did not produce enzyme induction Allmyr, Panagiotidis et al. (2009).
				**AhR, CAR, PXR:** Activated PXR, inhibited CAR1, had no effect on CAR2, and activated CAR3 Paul, Thompson et al. (2013). Modulated the expression level of AhR, CYP1A1, and CYP1B1 *in vitro* in mouse neocortical neurons Szychowski, Wnuk et al. (2016).
				**Additional Info:** In mice, induces hepatic steatohepatitis through mechanisms involving activating transcription factor 4, PPARα, and fibroblast growth factor 21 Yueh, He et al. (2020).
**Caffeine**; 58-08-2; 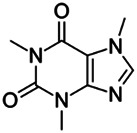 ; Stimulant found naturally in coffee and tea that is added to drinks and medicines; 194.191; −0.13	Inhibitor	NA	NA	**CYP1A1:** In REPS, 0.1 mM had no effect on enzyme activity, and 1 mM inhibited enzyme activity by ∼50% Tassaneeyakul, Birkett et al. (1993).
				**CYP1A2:** In PHH, 20–200 µM had no effect on mRNA, and a high 400 µM increased mRNA 2.3 × Vaynshteyn and Jeong (2012). Inhibited enzyme activity in REPS (inhibition of ∼15% at 0.1 mM and ∼70% at 1 mM) Tassaneeyakul, Birkett et al. (1993) and in a clinical study (600 mg increased the AUC of the CYP1A2 substrate, melatonin by 120%) Hartter, Nordmark et al. (2003).
				**AhR:** Did not activate AhR Vaynshteyn and Jeong (2012).
				**Additional Info:** Metabolised by CYP1A2 (>95% of its primary metabolism), CYP2C8/9, CYP2A6, CYP2E15, and **CYP3A4** Kot and Daniel (2008b), OECD (2020b), The Danish Centre on Endocrine Disrupters (2020), Thorn, Aklillu et al. (2012). In humans, caffeine metabolism has been used as a marker of CYP1A2 and CYP2A6 metabolism, and gender did not affect CYP1A2 or CYP2A6 mediated metabolism of caffeine Begas, Kouvaras et al. (2007). There are species differences between rat and human metabolism, but in both species, metabolism is mainly mediated by CYP1A2 Kot and Daniel (2008a). **Human Exposure Data Available:** Urine concentrations of caffeine and its metabolites in volunteers dosed orally with caffeine are available Begas, Kouvaras et al. (2007), Kim, Choi et al. (2019).
Part B. Candidate Selection Pool Chemicals Evaluated but not Selected				
**Fipronil**; 120068-37-3; 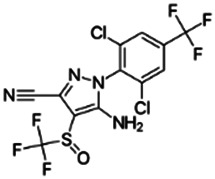 ; Broad-spectrum phenylpyrazole class insecticide	Inducer (+)	NA	Inducer (++)	**CYP1A1:** In HepaRG cells, 50 µM decreased mRNA and had no effect on protein Braeuning, Mentz et al. (2020), Schmidt, Lichtenstein et al. (2021), whereas, in PHH, 0.1-25 µM increased mRNA ≤50x and enzyme activity ≤2.5x (bell-shaped concentration response) Das, Cao et al. (2006), Mitchell, Dhammi et al. (2016).
				**CYP1A2:** In PHH, 0.1-25 µM had no effect on mRNA Das, Cao et al. (2006), Mitchell, Dhammi et al. (2016). In HepaRG cells, 50 µM had no effect on protein Braeuning, Mentz et al. (2020) **,** Schmidt, Lichtenstein et al. (2021). In REPS, 50 µM non-significantly increased enzyme activity ∼15% Usmani, Cho et al. (2006).
				**CYP2B6:** In PHH, 0.1-25 µM increased mRNA ≤3.5 × Das, Cao et al. (2006), Lawrie, Mitchell et al. (2020), Mitchell, Dhammi et al. (2016). In HepaRG cells, 50 µM had no effect on protein Schmidt, Lichtenstein et al. (2021).
				**CYP3A4:** In HepaRG cells, 50 µM increased protein 0.4 (log_2_)x [ = 1.3x] Braeuning, Mentz et al. (2020), Schmidt, Lichtenstein et al. (2021). In PHH, 0.1-25 µM increased mRNA ≤28x and protein and enzyme activity ≤4x (bell-shaped concentration response) Das, Cao et al. (2006), Hodgson and Rose (2007), Lawrie, Mitchell et al. (2020), Mitchell, Dhammi et al. (2016). In REPS, 50 µM had no effect on enzyme activity Usmani, Cho et al. (2006).
				**PXR:** Activated PXR Lemaire, Mnif et al. (2006).
**Ketoconazole**; 65277-42-1; 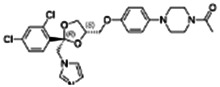 ; Fungicide (pharmaceutical); 437.148; 4.76	Inducer (++)	NA	No Effect or Inhibitor	**CYP1A1:** In HepG2 cells, 1–50 µM increased mRNA ≤350x, protein by an undescribed amount, and enzyme activity ≤4.5x, whereas, in PHH, 1–50 µM increased mRNA ≤10x, and had no effect on enzyme activity Novotna et al. (2014a). In Caco-2 cells, 4.7 µM moderately increased enzyme (EROD) activity (∼10 pmol/min/mg protein vs. 0 for the control) Sergent, Dupont et al. (2009). In HepG2 cells, 1–50 µM increased mRNA ≥10x, protein ≥4x and enzyme (EROD) activity ≤3 × Korashy, Shayeganpour et al. (2007).
				**CYP1A2:** In PHH, 1–50 µM increased mRNA ≤10x, and protein by an undefined amount Novotna et al. (2014a). In REPS, produced no Yim, Kim et al. (2020) or weak inhibition (∼20% at 40 μM and ∼50% at 120 µM) Emoto, Murase et al. (2003) of enzyme activity.
				**CYP2B6:** In REPs, inhibited enzyme activity with an IC_50_ of 3.18 µM Walsky, Astuccio et al. (2006).
				**CYP3A4:** In HepG2 cells and PHH, 1–50 µM increased mRNA ≤5x and protein by an undefined amount, but in HLM, potently inhibited enzyme activity with Ki’s of 0.27 and 2.28 µM Novotna et al. (2014b). In Caco-2 cells, 4.7 µM produced a non-significant ∼20% decrease in enzyme activity Sergent, Dupont et al. (2009).
				**AhR:** Activated and inhibited AhR Novotna et al. (2014a).
				**Other CYP Effects:** Inhibited CYP2C19, 11B1, 11B2, 11A1, and 17 Hu and Hartmann (2014). Potential enantiospecific effects were observed on microsomal CYP3A4 enzyme inhibition, but not on PXR agonism or CYP3A4 enzyme induction in the HepG2 cell bioassay Novotna et al. (2014b).
permethrin**; 52645-53-1; 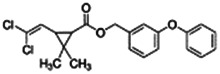 ; Pharmaceutical and insecticide (pyrethroid); 391.288; 7.15	No Effect	No Effect	NA	**CYP1A2:** In PHH, 100 µM had no effect on mRNA Das et al. (2008b). In REPS, 50 µM had no effect on enzyme activity Usmani, Cho et al. (2006).
				**CYP2B6:** In PHH, 100 µM had no effect on mRNA Das et al. (2008b).
				**CYP3A4:** In PHH, in one study, 100 µM had no effect on mRNA Das et al. (2008b), while in another, 10 µM increased mRNA ≤2 × Yang, Wang et al. (2009). In REPS, 50 µM inhibited enzyme activity ≤37% Usmani, Cho et al. (2006)
				**PXR:** Activated PXR Das et al. (2008b) **,** Yang, Wang et al. (2009).
				**Human Exposure Data:** Plasma and urine PK data from volunteers orally dosed with 0.1 mg/kg permethrin Ratelle, Côté et al. (2015), urine PK data from volunteers after dermal application National Research Council (1994), and urine PK data from agricultural workers after occupational exposure Ferland, Côté et al. (2015).
**Parathion**; 56-38-2 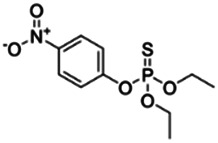 ; Organophosphate insecticide and acaricide; 291.261; 3.84	NA	Inducer (∼)	NA	**CYP1A1:** In HepG2 cells, 100/1000 µM increased mRNA ≤160 × Vrzal, Zenata et al. (2015).
				**CYP1A2:** In PHH,100/1000 µM increased mRNA ≤20 × Vrzal, Zenata et al. (2015). In REPS, inhibited enzyme activity with an IC_50_ of 0.8 µM Di Consiglio, Meneguz et al. (2005).
				**CYP2B6:** In PHH, 100/1000 µM increased mRNA ≤10 × Vrzal, Zenata et al. (2015).
				**CYP3A4:** In PHH, 100/1000 µM increased mRNA ≤40x and strongly increased protein Vrzal, Zenata et al. (2015). In REPS, inhibited enzyme activity with an IC_50_ of 5 µM Di Consiglio, Meneguz et al. (2005).
				**AhR, PXR:** Activated the AhR and PXR with bell shaped concentration responses Vrzal, Zenata et al. (2015).
**Pyrimethanil**; 53112-28-0; 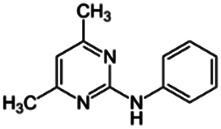 ; Broad spectrum fungicide; 199.252; ; 2.84	Inducer (∼)	NA	NA	**AhR:** Activated AhR with an EC_50_ of 4.6 µM Medjakovic, Zoechling et al. (2014); **Human Exposure Data:** Urine PK data from volunteers dosed with 0.17 mg/kg/day via the oral and dermal route, from an environmentally exposed general population cohort, and from an occupationally exposed horticulturist cohort Faniband, Ekman et al. (2019).
**Propetamphos**; 31218-83-4; 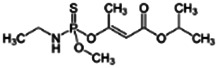 ; Organophosphate insecticide; 281.309; 1.61	NA	NA	NA	**Human Exposure Data:** Blood and urine PK data from orally and dermally dosed volunteers Garfitt, Jones et al. (2002).
**Tetrabrominated BPA (TBBPA)**; 79-94-7; 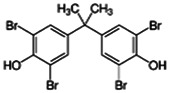 ; Flame retardant; 543.871; 7.29	NA	NA	Inducer (∼)	**CYP3A4:** In HepG2 cells, 10 µM increased mRNA ∼3 × Gramec Skledar, Tomasic et al. (2016).
				**AhR, PXR:** Activated PXR, and had no effect on AhR Gramec Skledar, Tomasic et al. (2016).
				**Other CYP Effects:** After *in vivo* dosing to rats, there was no significant effect on CYP3A1/3A3, CYP1A1/1A2 and CYP2B mRNA levels and enzyme activities Germer, Piersma et al. (2006).
				**Human Exposure Data:** After oral dosing, TBBPA has very low systemic bioavailability in humans and rats Schauer et al. (2006).
**Prochloraz**; 67747-09-5; 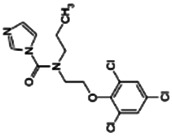 ; imidazole fungicide; 376.665; 3.98	NA	No Effect	Inducer (∼)	**CYP1A1:** In HepaRG cells, 80 µM increased mRNA ∼1.6x, and had no effect on protein Braeuning, Mentz et al. (2020) **,** Schmidt, Lichtenstein et al. (2021).
				**CYP1A2:** In HepaRG cells, 80 µM reduced protein ∼0.5 (log_2_)x [ = ∼1.4x] Schmidt, Lichtenstein et al. (2021).
				**CYP2B6:** In HepaRG cells, 80 µM had no effect on protein Schmidt, Lichtenstein et al. (2021).
				**CYP3A4:** In HepaRG cells, 80 µM increased protein ≤1.6 (log_2_)x [ = ≤3.0x] Braeuning, Mentz et al. (2020) **,** Schmidt, Lichtenstein et al. (2021).
				**Other CYP Effects:** Is a potent phenobarbital-type inducer of CYP enzyme activity in rats and mice EFSA (2011).
				**Additional Info:** It is an aromatase inhibitor, and has anti-androgenic and anti-estrogenic activity The Danish Centre on Endocrine Disrupters (2020). Its antiandrogenic action is produced by a dual mode: androgen receptor blocking and fetal steroidogenesis inhibition Vinggaard, Hass et al. (2006).
**Rotenone**; 83-79-4 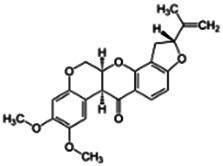 ; Naturally occurring isoflavone pesticide and piscicide; 394.417; 4.65	NA	NA	NA	**Other CYP Effects:** Metabolised via CYP3A4 and CYP2C19, but not via CYP2A6, 2C9, 2D6, 2E1 Caboni, Sherer et al. (2004) **,** OECD (2020a).
				**Human Exposure Data:** Concentrations in biological samples from a fatally poisoned girl De Wilde, Heyndrickx et al. (1986).
**Chlorpyrifos-methyl**; 5598-13-0; 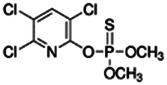 ; AChE Inhibitor Organophosphate Pesticide; 322.533; 3.71	Inducer (∼)	NA	NA	**AhR:** Activated AhR with an EC_50_ of 5.1 µM Medjakovic, Zoechling et al. (2014).

a Source Chemspider, (www.chemspider.com). Accessed 14 April 2022.

The selected set contains the following number of expected enzyme inducers: 7 x CYP1A1/1A2 (four low, two strong, one uncertain), 6 x CYP2B6 (two low, two moderate, two uncertain), and 8 x CYP3A4 (four low, two moderate, one strong, one uncertain). These will allow for a sufficient evaluation of the ability of the assay to accurately measure the activity of all four CYP isoforms that are covered by this assay. Additionally, the selected set also contains two expected inhibitors for CYP1A1, CYP2B6, and CYP3A4, which will allow also for an evaluation of the performance of the test method in detecting CYP enzyme inhibitors. For validation experiments, whilst it is considered good practice to include a proficiency chemical set that contains at least 25% of negative chemicals, unfortunately it was not possible to fully meet this criterion for this augmented chemical set, as sufficient data regarding no CYP activity was only available for two of the 23 candidate pool chemicals. Therefore, the augmentation chemical set contains two chemicals that are expected to have no effect on CYP1A1/1A2 activity, but no additional non pharmaceutical chemicals that are expected to have no effect on CYP2B6 or CYP3A4 activity. Following the additional chemical augmentation validation confirmatory testing, the relative potencies of the chemicals in Table 2 will be consolidated.

**TABLE 2:** Set of Proficiency Pharmaceutical Chemicals that have been Evaluated in Experiments Carried out by Bernasconi, Pelkonen et al., 2019 to Validate the *In Vitro* CYP Induction Assay in Primary Human Hepatocytes and HepaRG cells.

Chemical name CAS number Molecular weight (Dalton)^a^ Predicted LogP^a^	Structure	Pharmaceutical class	Evidence for the presence (yes) or lack (no) of human CYP induction activity in vitro or in vivo in clinical studies. Na = No data found/Available					
			CYP1A1/1A2		CYP2B6		CYP3A4	
			*In Vitro*	*In Vivo*	*In Vitro*	*In Vivo*	*In Vitro*	*In Vivo*
Omeprazole	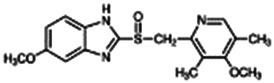	Proton-pump inhibitor	Yes Bernasconi, Pelkonen et al. (2019)	Yes Rost, Brosicke et al. (1994); Rost, Fuhr et al. (1999); Sarich, Kalhorn et al. (1997); Andersson, Rohss et al. (2001); Bernasconi, Pelkonen et al. (2019),^g^	Yes Bernasconi, Pelkonen et al. (2019) ^,^ ^c^	NA	No Bernasconi, Pelkonen et al. (2019)	No Rost, Brosicke et al. (1994); Andersson, Rohss et al. (2001); Zhou, Pant et al. (2018)
73590-58-6								
345.416								
2.17								
Carbamazepine	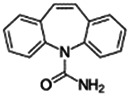	Anticonvulsant	Yes Bernasconi, Pelkonen et al. (2019) ^b^	Yes Parker, Pritchard et al. (1998); Lucas, Gilfillan et al. (1998); Oscarson, Zanger et al. (2006)	Yes Bernasconi, Pelkonen et al. (2019)	Yes Ketter, Jenkins et al. (1995); Ji, Damle et al. (2008)	Yes Bernasconi, Pelkonen et al. (2019)	Yes Moreland, Park et al. (1982); Crawford, Chadwick et al. (1990); Herman, Locatelli et al. (2006); Perucca, Hedges et al. (2004)
298-46-4								
236.269								
2.67								
Phenytoin Sodium	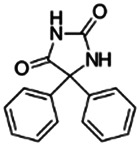	Anticonvulsant	Yes Bernasconi, Pelkonen et al. (2019)	Yes Wietholtz, Zysset et al. (1989); Miller, Cosgriff et al. (1984)	Yes Bernasconi, Pelkonen et al. (2019)	Yes Slattery, Kalhorn et al. (1996); Williams, Wainer et al. (1999)	Yes Bernasconi, Pelkonen et al. (2019)	Yes Werk, Macgee et al. (1964); Crawford, Chadwick et al. (1990)
630-93-3								
274.250								
2.29								
Penicillin G sodium	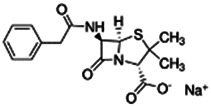	Antibiotic	No Bernasconi, Pelkonen et al. (2019)	NA	No Bernasconi, Pelkonen et al. (2019)	NA	No Bernasconi, Pelkonen et al. (2019)	NA
69-57-8								
356.372								
1.67								
Rifabutin	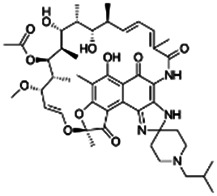	Antimicrobial	Yes Bernasconi, Pelkonen et al. (2019)	No Gillum, Sesler et al. (1996)	Yes Bernasconi, Pelkonen et al. (2019)	NA	Yes Bernasconi, Pelkonen et al. (2019),Horita and Doi (2014)	Yes Barditch-Crovo, Trapnell et al. (1999)
72559-06-9								
847								
3.45								
Sulfinpyrazone	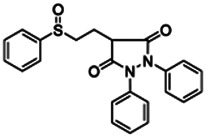	Uricosuric	Yes Bernasconi, Pelkonen et al. (2019) ^b^	Yes Birkett, Miners et al. (1983)	Yes Bernasconi, Pelkonen et al. (2019)	NA	Yes Bernasconi, Pelkonen et al. (2019),Pichard, Fabre et al. (1990)	Yes Birkett, Miners et al. (1983); Wing, Miners et al. (1985); Walter, Staiger et al. (1982); Staiger, Schlicht et al. (1983)
57-96-5								
404.482								
2.3								
Bosentan hydrate	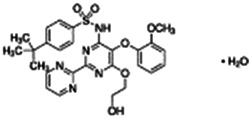	Endothelin Receptor Antagonist	Yes Bernasconi, Pelkonen et al. (2019) ^b^	NA	Yes Bernasconi, Pelkonen et al. (2019)	NA	Yes Bernasconi, Pelkonen et al. (2019)	Yes Weber et al. (1999a); Weber et al. (1999b); van Giersbergen et al. (2002a); van Giersbergen et al. (2002b); Dingemanse and van Giersbergen (2004)
157212-55-0								
569.629								
1.15								
Artemisinin	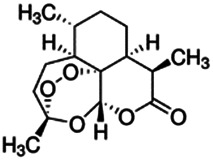	Anti-malarial	No Bernasconi, Pelkonen et al. (2019)	NA	Yes Bernasconi, Pelkonen et al. (2019)	Yes Simonsson, Jansson et al. (2003); Elsherbiny, Asimus et al. (2008)	Yes Bernasconi, Pelkonen et al. (2019) ^d^	Yes/No^19,^ ^h^
63968-64-9								
282.332								
2.27								
Efavirenz	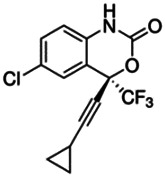	Anti-retroviral	No Bernasconi, Pelkonen et al. (2019)	NA	Yes Bernasconi, Pelkonen et al. (2019)	Yes Robertson, Maldarelli et al. (2008); Ngaimisi, Mugusi et al. (2010)	Yes Bernasconi, Pelkonen et al. (2019)	Yes Mouly, Lown et al. (2002); Fellay, Marzolini et al. (2005)
154598-52-4A								
315.675								
4.84								
Rifampicin	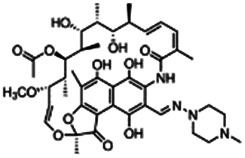	Antibiotic	Yes Bernasconi, Pelkonen et al. (2019) ^b^	Yes Robson, Miners et al. (1984); Wietholtz, Zysset et al. (1995); Backman, Granfors et al. (2006)	Yes Bernasconi, Pelkonen et al. (2019)	Yes Lopez-Cortes, Ruiz-Valderas et al. (2002); Loboz, Gross et al. (2006)	Yes Bernasconi, Pelkonen et al. (2019)	Yes Ohnhaus and Park (1979); Barditch-Crovo, Trapnell et al. (1999); Lin (2006); Kanebratt, Diczfalusy et al. (2008)
13292-46-1								
822.940								
1.09								
Metoprolol	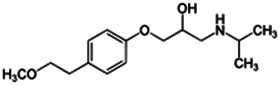	β1 receptor blocker	No Bernasconi, Pelkonen et al. (2019) ^e^	NA	No Bernasconi, Pelkonen et al. (2019) ^e^	NA	No Bernasconi, Pelkonen et al. (2019) ^e^	NA
51384-51-1								
267.364								
1.79								
Sotalol	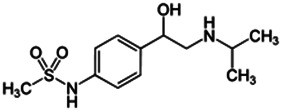	Non-selective *β* receptor blocker	No Bernasconi, Pelkonen et al. (2019)	NA^f^	No Bernasconi, Pelkonen et al. (2019)	NA^f^	No Bernasconi, Pelkonen et al. (2019)	NA^f^
959-24-0								
272.364								
0.32								

a Source Chemspider, (www.chemspider.com). Accessed 14 April 2022.

Key.

b = For CYP1A2, carbamazepine, sulfinpyrazone, bosentan, and rifampicin were positive in HepaRG, cells and negative in PHH, cells.

c = For CYP2B6, omeprazole was positive in PHH, cells, and negative in HepaRG, cells.

d = For CYP3A4, artemisinin was positive in PHH, cells, and negative in HepaRG, cells.

e = A small fraction of metoprolol is metabolised *in vitro* by CYP3A4, CYP2B6 and CYP2C9 (Berger, Bachmann et al., 2018), but there is no evidence for CYP, induction.

f = Sotalol is considered unlikely to induce CYP, enzymes, due to its PK, characteristics (Yamreudeewong, DeBisschop et al., 2003).

g = Omeprazole induced CYP1A2 in humans, but only at high non-clinically relevant doses.

h = For artemisinin, CYP3A4 induction was detected in Asimus, Elsherbiny et al., 2007; while in Svensson, Ashton et al., 1998 no induction was observed.

Validation data for the test method has previously been generated for the 12 pharmaceutical proficiency chemicals shown in Table 2, and *in silico* chemical space evaluations are reported to demonstrate that the structures of the latter pharmaceuticals are representative of EU REACH (https://echa.europa.eu/regulations/reach/legislation), Drugbank, and Tox21 listed chemicals (Bernasconi, Pelkonen et al., 2019). Overall, the total set of 25 proficiency chemicals (including the 12 pharmaceuticals and the proposed 13 additional industrial, pesticidal, and food additive chemicals) is considered to be sufficiently diverse and representative of OECD TG Programme relevant chemical classes to facilitate draft TG approval at the OECD.

### Applications of *In Vitro* Metabolism Data Including CYP Induction Data in Chemical Hazard Assessment: Meeting the outstanding needs to achieve OECD test method adoption

#### OECD Human *in vitro* Metabolism Test Method Development Needs

PK, including absorption, distribution, metabolism, and excretion (ADME) play a key role in determining *in vivo* exposure to a parent chemical and its metabolites after dosing, and PK data is used extensively in the design and interpretation of toxicological assessments of test chemicals. Currently, toxicokinetic ADME data for single and repeated dose *in vivo* studies (OECD 2010) is commonly generated and used in Europe for high tonnage industrial chemicals and for pesticides and worldwide for pharmaceuticals (ICH S3A, 1994). Relevant data from several *in vitro* ADME assays, including, for example, Caco-2 cell permeability assays measuring absorption potential; transporter protein substrate/inhibition assays which provide data on distribution, excretion, and PK interactions; S9 and microsome addition and *in vitro* metabolism assay data can contribute on a weight of evidence basis to the toxicological hazard assessment. However, when the data is generated according to an accepted OECD TG as part of the Mutual Acceptance of Data agreement, this data can be submitted without additional testing to many OECD regulatory jurisdictions. The process of validation is intended to establish the relevance, reproducibility and reliability of a test method for a specific regulatory purpose (OECD 2005) and gives much greater confidence in the reliability of the test data generated.

That there is an urgent need to develop new OECD TGs for these *in vitro* test method is well established at the OECD (Jacobs, Janssens et al., 2008; OECD 2008; Jacobs, Laws et al., 2013; Bernasconi, Pelkonen et al., 2019), as data from these assays could be used alongside standard *in vivo* PK data in a complimentary fashion to aid toxicological assessments. Importantly, several quantitative *in vitro* to *in vivo* extrapolation (QIVIVE) and *in silico* physiology-based pharmacokinetic (PBPK) models have been shown to be capable of accurately predicting *in vivo* PK parameters from *in vitro* data (OECD, 2021; Tsaioun, Blaauboer et al., 2016), and it is hoped that in the future QIVIVE and PBPK approaches could replace certain types of *in vivo* PK data, which would allow for a significant reduction in animal use.

With regards to metabolism, the availability of additional validated *in vitro* assays capable of generating human relevant metabolic profiling and CYP induction/inhibition data would be particularly valuable. Animal relevant metabolic profile data from non-human *in vivo* studies is often available. However, to date, there are no adopted TGs available for *in vitro* test method that produce human metabolic profile data, which hampers efforts focused on determining the relevance of animal metabolite data to humans. Moreover, for results from *in vitro* toxicity assays to accurately predict potential *in vivo* toxicity, it is essential that the concentrations of parent chemical/metabolites that are tested *in vitro* are representative of *in vivo* levels, and, for this reason, metabolic transformation steps are included in many *in vitro* toxicity assays, including all of the OECD TG *in vitro* genotoxicity test method. At the moment, there are, however, a number of *in vitro* OECD TGs that lack appropriate (pre-)incubation steps to account for *in vivo* metabolism, including, for example, all of the Level 2 *in vitro* mechanistic human cell based test method specified in the current OECD Endocrine Disruptor Guidance Document 150 (OECD 2018). Moreover, for many chemicals, there are significant differences in metabolism between rats and humans, and only rat S9 microsomes are used to produce metabolic transformation in the OECD genotoxicity TGs. In a recent European Food Safety Authority (EFSA) scientific panel opinion paper relating to the toxicological testing of pesticides, the use of *in vitro* human metabolite data is recommended to identify any potential human relevant metabolites that had not been adequately tested in non-human toxicological studies (EFSA 2021).

### Applications of *in vitro* Human CYP Induction Data in Chemical Hazard Assessment

**FIGURE 1:**
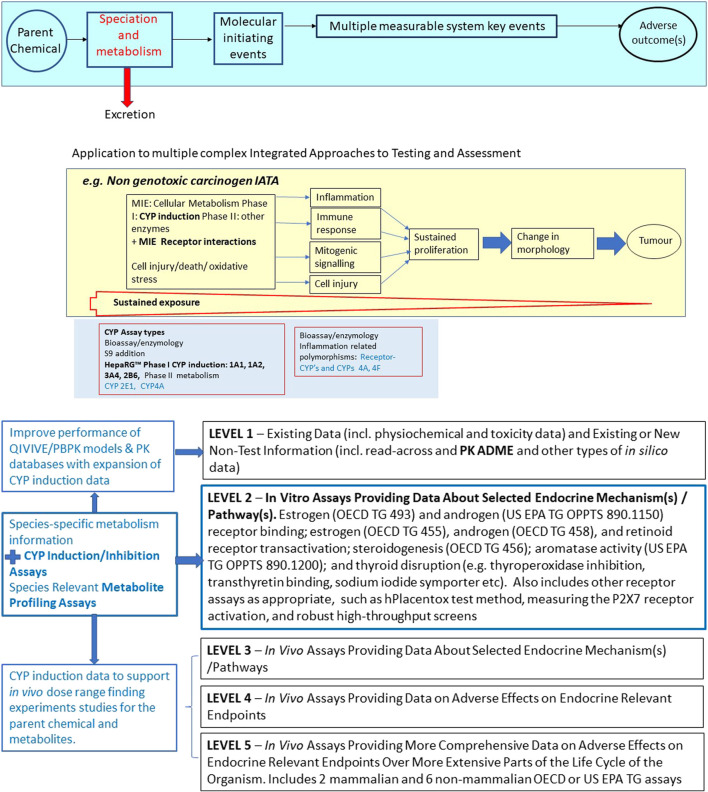
Selected illustrations of regulatory applications of *in vitro* metabolism test method/systems falling within the OECD TG Programme. **panel (A)** Introduction of metabolism *in vitro* testing at Level 2 of the OECD Conceptual Framework for Testing and Assessment of Endocrine Disrupting Chemicals (EDCs).

CYP induction data can indicate whether and to what extent a chemical is likely to undergo CYP-mediated metabolism, and results showing significant CYP induction could be used as an indicator that (pre-)incubation steps to account for *in vivo* metabolism should be included in any toxicity assays lacking metabolic competence. In relevant situations, CYP induction and inhibition data could facilitate the selection of optimal *in vivo* test chemical doses for human and other animal studies, and also indicate the possible involvement of CYP metabolism/metabolites in adverse or PK effects. Perturbations in the levels of endogenous chemicals that are metabolised by CYPs are associated with several adverse effects, and CYP induction data could also be used to support the contribution of CYP-mediated mechanisms in adverse outcome pathways and IATAs as shown in Figure 1B. Furthermore, the performance of a number of QIVIVE/PBPK models and PK databases such as MetaPath (Kolanczyk, Schmieder et al., 2012) would be substantially expanded by the incorporation of available CYP induction data. It would be particularly beneficial to generate CYP data for food chemical classes for example, as in Europe, little mammalian *in vivo* data is available for these.

Moreover, CYP induction and inhibition data are currently commonly used to predict potential CYP mediated clinical PK drug interactions for pharmaceuticals (EMA 2012; US FDA, 2020) and the availability and use of a validated and adopted OECD CYP induction test method, that will fall under the Mutual Acceptance of Data agreement, will therefore also be valuable for drug discovery and regulatory application for pharmaceutical CYP drug interaction evaluations.

### Examples of Applications of *in vitro* Human CYP Induction Data for IATAs

Relevant *in vitro* metabolism data would greatly benefit several specific IATAs, including the developmental neurotoxicity (Bal-Price, Crofton et al., 2015) and non-genotoxic carcinogenicity (Jacobs, Colacci et al., 2020) IATAs currently under development, and the OECD Conceptual Framework for Endocrine Disruptors (updated OECD 2018). With respect to the latter, as proposed in 2013 (Jacobs, Laws et al., 2013), *in vitro* metabolism test method can be added at level 2 of the Endocrine Disruptor Conceptual Framework as shown in Figure 1A,B below, but will also have great utility in informing all levels of the Conceptual Framework. This will begin to accommodate the additional *in vitro* assay flexibility needs for the regulatory identification of endocrine disruptors (Solecki, Kortenkamp et al., 2017), by filling the metabolism translational gap between *in vitro* level 2 assays and the WHO definition of endocrine disruptor, as “an exogenous substance or mixture that alters the function(s) of the endocrine system and consequently causes adverse effects in an intact organism, or its progeny, or (sub) populations”. Quoting from the consensus paper ‘a) *Alterations of the function of the endocrine system may arise from interaction with hormone receptors, changes in circulating levels of the hormone, and from the impact of chemical(s) on hormone synthesis, transport,* ***metabolism*** *and other factors*’*. c*) *The term “intact organism” is understood to mean that the effect would occur in vivo, either observable in a test animal system, epidemiologically or clinically. However, it does not necessarily mean that the adverse effect has to be demonstrated in an intact test animal,* ***but may be shown in adequately validated alternative test systems predictive of adverse effects in humans*** *and/or wildlife*‘ (Solecki, Kortenkamp et al., 2017).

In addition to mediating detoxification, CAR, PXR and AhR have been implicated in the regulation of a broader range of physiological functions (Kretschmer and Baldwin 2005; Wang and Tompkins 2008; Yi, Fashe et al., 2020), where dysregulation can lead to adverse effects (Hakkola, Bernasconi et al., 2018) and receptor and CYP induction have well documented roles for instance in inflammation (Christmas, 2015; Rubin, Janefeldt et al., 2015), cholestasis, steatosis (Gomez-Lechon, Jover et al., 2009), hepatotoxicity (Woolbright and Jaeschke 2015), carcinogenesis (De Mattia, Cecchin et al., 2016; Pondugula, Pavek et al., 2016; Fucic, Guszak et al., 2017), and thyroid disruption (OECD 2006; OECD 2014). Thus, in these cases, induction of specified CYP enzymes may serve as a biomarker for key events associated with adverse health effects. In addition, the use of CYP induction data, in combination with assays that can address foetal and early life chemical exposure mediated via the placenta, such as the hPlacentox human placental JEG-3 cell line model (Rat, Olivier et al., 2017; Olivier, Wakx et al., 2021) will assist regulatory *in vitro* tool development, reducing metabolism uncertainties and improving the evidence base to address ‘*disruption of the programming role of hormones during prenatal and postnatal development* [that] *can cause adverse effects that do not become evident until later in life*’ (Solecki, Kortenkamp et al., 2017). CYP3A induction increases by approximately 2-fold during pregnancy, and has been shown to be mediated by cortisol plasma concentrations during pregnancy (Sachar, Kelly et al., 2019).

CYP induction testing is a critical initial testing aspect in IATAs such as that for non-genotoxic carcinogenicity (Figure 1B).

The HepaRG CYP enzyme induction test method as validated thus far can address CYP1A, 3A4 and 2B6, as primary targets. Looking forward, assays for additional CYP isoforms will be needed for specific IATAs, as identified in Figure 1B, and for metabolism evaluations for chemicals that are metabolised by other inducible CYPs. For instance, an assay for CYP2E1 is being assessed within the OECD non-genotoxic carcinogenicity IATA expert group. Furthermore, among the xenobiotic-metabolizing CYPs, CYP2D6 results in a large contribution of genetic variation to the interindividual variation in CYP enzyme activity (Ingelman-Sundberg, Sim et al., 2007), and an FDA guideline on *in vitro* drug interaction testing of pharmaceuticals recommends also evaluating induction of CYP2C8, 2C9, 2C19 (US FDA, 2020). Thus, while inclusion of additional CYP enzymes (such as CYP2D6) and contribution of nuclear receptors such as the glucocorticoid receptor (GR) which induces CYP2D6 (Farooq, Kelly et al., 2016), and PPARs (which induce CYP4A, Kliewer, Lehmann et al., 1999, Waxman, 1999) would be useful to explore in the near future, the scope of this study was to augment the chemical applicability domain of the validated draft TG without *de novo* validation of additional targets.

It is noted that GR is functional in the HepaRG cell line (Hart, Li et al., 2010; Farooq, Kelly et al., 2016; Sachar, Kelly et al., 2019) and thus subsequent additional testing with glucocorticoids (such as prednisone, prednisolone, cortisone, corticosterone, dexamethasone, betamethasone, triamcinolone, 6-methylprednisolone, 21-deoxy cortisol, deflazacort and hydrocortisol) could be run in the assay to develop further model applications, to ascertain glucocorticoid activity via GR, especially when running the CYP HepaRG assay in relation to *in vitro* GR transactivation and placental models, or for inclusion of such information in a metabolic disruption IATA.

Moving beyond molecular initiating events, to subsequent key events, there will need to be specific considerations for the direct and indirect CYP activity in relation to inflammation for example, for carcinogenicity.

### Potential of the HepaRG Cell System to Be Further Developed for Additional Metabolism Relevant Endpoints

Following successful adoption and application of the HepaRG CYP enzyme induction test method, it would be useful to develop complementary components of *in vitro* human metabolism systems into OECD TGs, including induction/inhibition assays for additional CYP isoforms, and assays for Phase II metabolism, metabolite profiling, and metabolic transformation pre-incubation steps (as discussed above). The use of human microsomes and/or S9 mixes, or cryopreserved primary human hepatocytes (PHH) have utility in research and drug discovery, but for chemical hazard assessment purposes, cryopreserved PHH with very wide ranging variability in responses, had reproducibility issues in a validation exercise (Bernasconi, Pelkonen et al., 2019), and following OECD peer review, were considered too variable for TG development. There are also (unknown) viral transmission concerns with the use of primary human tissues in routine chemical testing.

The HepaRG CYP enzyme induction test method was agreed by the OECD member countries to be the best (longer term) option to take forward for regulatory use in 2008 (Jacobs, Janssens et al., 2008; OECD 2008; Jacobs, Laws et al., 2013), and following successful validation with pharmaceuticals (Bernasconi, Pelkonen et al., 2019) it is the most ready and reliable system available, and superior in validation performance to cryopreserved hepatocytes. A variety of additional non validated *in vitro* human liver on a chip and 3D liver organoid systems are currently in development, but the complexity, between batch variability, and lack of validation data for these models (Telles-Silva, Pacheco et al., 2022) mean that they are not ready for test guideline development at present. However available literature evidence indicates that HepaRG cells are well suited to all of these applications, as mRNA for many key proteins involved in xenobiotic metabolism are expressed in differentiated HepaRG cells, including xenobiotic sensing nuclear receptors (AhR, PXR, CAR, and PPAR-α), CYPs (CYP1A1, 1A2, 2A6, 2B6, 2C8, 2C9, 2C19, 2D6, 2E1, 3A4, 3A5, 3A7), other Phase I metabolic enzymes [including various isoforms of alcohol dehydrogenase, aldehyde dehydrogenase, and flavin-containing monooxygenase], and Phase II metabolic enzymes [including various isoforms of glutathione S-transferase, UDP-glucuronosyltransferase, N-acetyltransferase, and sulfotransferase] (Aninat, Piton et al., 2006; Guillouzo, Corlu et al., 2007; Josse, Aninat et al., 2008; Antherieu, Chesne et al., 2010; Hart, Li et al., 2010). Enzymatic activity for CYP1A1/1A2, 2B6, 2C8, 2C9, 2C19, 2D6, 2E1, and 3A4 has also been confirmed to be present (Aninat, Piton et al., 2006; Josse, Aninat et al., 2008; Antherieu, Chesne et al., 2010; Lubberstedt, Muller-Vieira et al., 2011). Furthermore, chemical-induced induction and inhibition of CYP1A1/1A2, 2A6, 2B6, 2C8, 2C9, 2C19, 2D6, 3A4 has been demonstrated in HepaRG cells (Aninat, Piton et al., 2006; Josse, Aninat et al., 2008; Kanebratt and Andersson 2008; Turpeinen, Tolonen et al., 2009; Antherieu, Chesne et al., 2010; Yajima, Uno et al., 2014). The metabolic profile data generated for several chemicals in HepaRG cells have also been shown to be equivalent to that produced using PHH, including aflatoxin B1 and acetaminophen (Aninat, Piton et al., 2006) but without the inherent biological variability observed with different batches of PHH. Intrinsic clearance values generated for a large number of reference drugs in HepaRG cells have also been shown to be equivalent to the values generated in PHH (Lubberstedt, Muller-Vieira et al., 2011; Zanelli, Caradonna et al., 2012). In the future a potentially promising approach to explore would therefore be to develop the HepaRG cell system into an all-in-one system/assay providing all the desired abovementioned *in vitro* metabolic functionalities.

For now however, it is really important to first address the outstanding steps required to enable the successful adoption of the HepaRG CYP enzyme induction test method as a TG, and the work described herein provides the essential concrete chemical selection and applications step required to enable international TG progress within an immediate timeframe. Following the planned additional validation experiments with this candidate chemical selection list there will be an evidence basis upon which to refine the chemical list for use as additional proficiency chemicals for the HepaRG CYP enzyme induction test method, and to do any further analyses that may be warranted.

## 4 Conclusion

Overall, it is apparent that the availability of CYP induction data would significantly aid the toxicological assessment of chemicals, and our ongoing work to augment the chemical applicability domain as an extension to the validation of the CYP enzyme induction HepaRG test method is a requirement for the (near) future approval of this test method, at the OECD.

Here we have also given some examples of immediate applications of the assay for the OECD Conceptual Framework for Endocrine Disruptors, and for the OECD IATA for non-genotoxic carcinogens. There will also be necessary applications to other complex human health IATAs, including that for thyroid disruption (OECD, 2014), and metabolic disruption (Legler, Zalko et al., 2020).

## Abbreviations

AChE, Acetylcholinesterase; ADME, Absorption, Distribution, Metabolism, and Excretion; AhR, Aryl Hydrocarbon Receptor; B[a]P, Benzo-[a]-Pyrene; BPA, Bisphenol A; CAR, Constitutive Androstane Receptor; CYP, Cytochrome P450; DEET, N,N-Diethyl-m-Toluamide; DHMB, 2,3-Dihydroxy-4-Methoxybenzaldehyde; EDCs, Endocrine Disrupting Chemicals; EFSA, European Food Safety Authority; EROD, Ethoxyresorufin-O-Deethylase; HLM, Human Liver Microsomes; IATA, Integrated Approach to Testing and Assessment; ICH, International Conference on Harmonisation of Technical Requirements for Registration of Pharmaceuticals for Human Use; LC-MS, Liquid Chromatography-Mass Spectrometry; LOAEL, Lowest Observed Adverse Effect Level; MIE, Molecular Initiation Event; NAM, New Approach Methodologies; OECD, Organisation for Economic Cooperation and Development; PBPK, Physiology-Based Pharmacokinetic; PFOA, Perfluorooctanoic Acid; PHH, Primary Human Hepatocytes; PK, Pharmacokinetics; POPs, Persistent Organic Pollutants; PPARs, Peroxisome Proliferator Activated Receptors; PXR, Pregnane X Receptor; QIVIVE, Quantitative *In Vitro* to *In Vivo* Extrapolation; REPS, Recombinant Enzyme Preparations; TBBPA, Tetra Brominated Bisphenol A; TG, OECD Test Guideline; WNT, OECD Working Group of National Coordinators of the Test Guideline Programme.
